# The feasibility of new HPV/DNA test as a primary cervical cancer screening method among 35- years- old ever-married women in Kalutara district; a cross-sectional study

**DOI:** 10.1186/s12889-021-10190-4

**Published:** 2021-01-13

**Authors:** K. C. M. Perera, N. Mapitigama, HTCS Abeysena

**Affiliations:** 1 Ministry of Health, Colombo, Sri Lanka; 2 Family Health Bureau, Colombo, Sri Lanka; 3 University of Kelaniya, Kelaniya, Sri Lanka

**Keywords:** Cervical cancer, Screening, HPV/DNA test, Implementation, Feasibility

## Abstract

**Background:**

Cervical cancer is the second commonest female cancer in Sri Lanka. Two major drawbacks of the present cervical cancer screening programme are the suboptimal sensitivity of the pap smear and the low coverage. The objective of the study is to determine the feasibility of a new HPV/DNA test among 35 -years -old ever-married women in a district of Sri Lanka.

**Method:**

A community based descriptive cross-sectional study was conducted from 1^st^of July 2018 to 30th November 2018 in the public health divisions called Medical Officer of Health (MOH) areas of Kalutara district. The study population is comprised of ever-married women 35 years of age. Three women from each cluster (*n* = 413) were selected by consecutive sampling. A total of 918 women were recruited. HPV/DNA cervical specimen collection (*n* = 822) was carried out. Cervical specimens were tested by two cytoscreeners with the cobas 4800 PCR based screening machine. Clients’ perceptions and prevalence were assessed. The follow-up of women with positive HPV/DNA screening results was carried out. The operational and technical feasibility of the screening test were assessed. Data entry was done by using the statistical package IBM SPSS version 20.

**Results:**

Overall response rate was 91.1% (*n* = 836). Clients’ perception was highly positive for HPV/DNA screening test procedure (99.9%, *n* = 821) and 99.6% (*n* = 819) of clients had mentioned that the HPV/DNA screening test is worthwhile to be incorporated into the National Cervical Cancer Screening program. The prevalence of HPV was 6.2% (95%CI: 6.18–6.22%). The coverage of the HPV/DNA screening was 89.5%(*n* = 822). Invalid results reported were 0.12% (*n* = 1). The percentage of HPV/DNA test positive women who underwent pap test within 3 months of the initial screening was 100% (*n* = 51), while the percentage of women who attempted to get a colposcopy within the 1 month of referral was 86.7% (*n* = 13).

**Conclusions:**

HPV/DNA test implementation as a primary cervical cancer screening method is feasible among the 35- year age cohort of ever- married women in Kalutara district. It is necessary to further attempt alternative methods of cobas 4800 HPV/DNA test, which would be much suitable for resource-limited settings.

**Supplementary Information:**

The online version contains supplementary material available at 10.1186/s12889-021-10190-4.

## Background

Cervical cancer is the 2nd leading cause of female cancer in Sri Lanka and women at risk for cervical cancer are more than 8.4 million [1]. In Sri Lanka, annually 1136 new cases of cervical cancers are diagnosed and 643 women have died from the disease according to the estimates of 2018 [1].

Cervical cancer is virtually associated with human papillomavirus infection (HPV) but HPV infection does not always cause cervical cancer. Most women even infected with high-risk HPV types never develop cervical cancer, as most of this infection regardless of HPV type is short-lived and the body eliminates them spontaneously in ≤2 years. A small percentage of women with high-risk HPV infection can have persistent infection and progress into pre-cancer and even fewer women will progress to have invasive cancer [2].

More than a hundred and fifty HPV genotypes have been identified to date and nearly 60 different types of them are known to infect the human genital tract including cervix uteri. There are carcinogenic and non-carcinogenic genotypes and 10–15 carcinogenic genotypes are mainly associated with cervical cancer [3]. Some carcinogenic genotypes are classified as “high risk” (16,18,31,33,35,39,45,51,52,56,58 and 59) as there is an evidence of increased risk association between HPV infection and cervical cancer [4]. Compared to other carcinogenic genotypes of HPV infection, serotypes 16 and 18 have 190.3 times increased risk of developing cervical cancer [5]. Besides, HPV serotypes 26, 53, 66, 68, and 72 are considered as possible carcinogens but their role related to cervical carcinogenesis is unclear [6].

Cervical cancer screening is a secondary preventive strategy for the early detection of abnormal tissues from the cervix. In 1996, the concept of the Well Woman Clinic (WWC) programme was introduced to Sri Lanka and cervical cancer screening through WWCs was based on only visual inspection of the cervix. Subsequently, in 1998 cervical cancer screening based on visual inspection of the cervix with pap smear was included in WWC services.

The mean age of marriage for Sri Lankan female is 23.9 years [7]. Persistent HPV infection may progress into cervical precancerous lesions called Cervical Intraepithelial Neoplasia (CIN) within 10–20 years. Therefore the target age cohort for cervical cancer screening in Sri Lanka was taken as the age of 35 years (23.9 + 10 ~ 35 years) and the routine screening was carried out every 5 years between 35 and 60 years of women.

Nearly after 20 years of existence of the cervical cancer screening program, in contrast to the vigorous preventive measures, there is no marked reduction of incidence, of cervical cancer in Sri Lanka [1, 8] due to low sensitivity of the pap smear/conventional cytology (53%) [9] to detect CIN and low annual National coverage of the target age cohort women (35 years) by the screening programme [10]. In 2017, the coverage of the National cervical cancer screening programme was 53.3%, where the accepted target coverage is 80% [9]. Therefore, the cervical cancer prevention program needs to be reviewed with special attention.

HPV/DNA test is used to detect cervicovaginal HPV infection in women and the sensitivity of the test to detect CIN II viral load is 92.9% [11]. The objective of the study is to determine the feasibility of a new HPV/DNA screening test among 35- year age cohort of ever- married women in a district of Sri Lanka.

## Methods

A community based descriptive cross-sectional study was conducted from 1st of July 2018 to 30th November 2018. The study population comprised of ever-married women 35 years of age in public health divisions called Medical Officer of Health (MOH) areas of Kalutara district. Women with; diagnosed invasive cervical cancer, pregnancy, ≤ 3 months in the postpartum period, hysterectomy, per vaginal bleeding, active infection at the time of examination evidenced by medical records or by visual inspection, currently on treatment for HPV infection, diagnosed physical or mental retardation or disease status and women who did not reside within the district continuously for ≥3 months before the date of the survey were excluded from the study at the field and clinic settings.

For the calculation of the sample size to assess the present prevalence, we assumed that the expected prevalence of HPV was 3.3% [12] and the degree of accuracy desired specified as 0.016 (3.3/100 × 2) [13]. Therefore we needed 479 women. To account for the cluster effect, it was necessary to adjust the required sample size by having it multiplied by the design effect, which was taken as 1.1. Further adjustment to the sample size was made by considering the previous year’s WWC non-response rate (42.4%) in the Kalutara district [10] and the final required sample size was 915.

A Public Health Midwife (PHM) area was taken as a cluster. In the district, there are 413 PHM areas. Eligible Families Register/s was the sampling frame. Three women were selected from each cluster from the list of 35- year age cohort ever-married women population prepared from relevant area Eligible Families Register/s according to the ascending order of register numbers. We selected the first subject from the list by using a lot method and then two more subjects were selected forward and consecutively. A total number of 918 women were recruited to the study after applying possible exclusion criteria in the field setting. The number of women who approached clinics for HPV/DNA specimen collection was 836 (91.1%), therefore the number of non-respondents was 82 (8.9%). After applying the rest of the exclusion criteria at the clinic setting 822 (89.5%) women were subjected to a specimen collection.

Information regarding socio-demographic characteristics and client perceptions for new HPV/DNA test implementation were gathered by using an interviewer-administered questionnaire, developed for this study and attached as an additional file 1 (part 1). Face and content validity were assessed by the panel of experts during the process of questionnaire development. The questionnaire was pre-tested with 15 women at a WWC to identify deficiencies and for necessary modifications.

Public Health Midwives (PHMM) were uniformly trained to locate households. A two-day training session was conducted for research assistants (RA) (five pre-intern medical graduates) to familiarize them with proceedings. They were uniformly trained to administer an interviewer- administered questionnaire with socio-demographic characteristics of participants and it was administered at a separate and neutral place within the house. Age was calculated using the date of birth by recall or by using the National Identity Card and approximated to the last completed year. Once a questionnaire was completed, the participant was given a clinic appointment card with a reference number and invited to attend the clinic.

Staff training was done to collect cervical HPV/DNA specimens. Videos and guidelines were used for staff training. Instruction regarding accurate numbering of specimens, completion of specimen request forms, and preparation for transport were also included in the training sessions. Two cyto-screeners were uniformly trained for specimen barcoding, to handle the machine and report writing to ensure the quality of performance. Colposcopists too were uniformly trained to ensure the quality of performance.

HPV/DNA cervical specimen(*n* = 822) collection was carried out by Medical Officers (MOO) or Public Health Nursing Sisters (PHNSS) at WWCs. Cusco’s speculum was inserted to visualize the cervix before obtaining the HPV/DNA cervical specimen. HPV/DNA specimens obtained from the cervix using special broom-like devices were separately placed into HPV/DNA specimen collection containers with cell collection media/thinprep (20 ml). Cervical specimens were packed and transported to the laboratory District General Hospital (DGH), Kalutara. Prepared guidelines were strictly adhered to during data collection, barcoding, and preparation for transport and attached as an additional file 2. Cervical specimens were screened at the laboratory by well trained two cytoscreeners with cobas 4800 HPV/DNA automated PCR machine, which consists of cobas 4800x instrument and cobas analyzer. Cobas 4800 HPV/DNA screening machine was included several quality control mechanisms such as internal quality control, external quality control, and contamination control. The test sensitivity and specificity to detect CIN II is 92.9 and 71% respectively [11]. It detects 14 high risk carcinogenic HPV serotypes, such as; 16, 18 and 12 pooled high risk (31, 33, 35, 39, 45, 51, 52, 56, 58, 59, 66 and 68).

Variables used to assess the feasibility were; client perceptions for the new HPV/DNA test and it’s positive follow-up, the overall prevalence of cervicovaginal HPV infection and it’s subgroup analysis, HPV/DNA screen positive follow-up results according to the algorithm of Asia Oceania guidelines for the implementation of programs for cervical cancer prevention and control [14] and operational and technical feasibility of HPV/DNA screening implementation.

Client perceptions for the new HPV/DNA test and it’s positive follow-up were gathered by using an interviewer-administered questionnaire (part 2 of the additional file 1) following cervical HPV/DNA specimen collection (*n* = 822) at community WWCs (*n* = 89) in Kalutara district by RAs. The descriptive longitudinal study design was used to describe the follow-up of women with positive HPV/DNA screening results. All HPV/DNA positives (*n* = 51) were subjected to pap smear at community WWCs and pap screening was carried out at District General Hospital, Kalutara, and National Institute of Health Science (NIHS), Kalutara. HPV/DNA positive and cytology result ≥ Atypical Squamous Cells of Undetermined Significance (ASCUS) were referred to colposcopy either at Kethumathie Maternity Hospital, Panadura, or Family Health Bureau, Colombo.

A cross-sectional descriptive study design was used to assess the operational and technical feasibility of HPV/DNA screening implementation. Operational feasibility of the HPV/DNA screening was calculated by using the following feasibility indicators; HPV/DNA coverage, number of invalid results reported, treatment adherence percentage of HPV/DNA + ves up to pap screening within 3 months of initial screening, and colposcopy attempting percentage within 1 month of referral. Technical feasibility of the HPV/DNA screening was calculated by using the following feasibility indicators; duration of time between specimen collection and transport to the laboratory, duration of time between specimen collection and report delivery to the relevant MOH office, the mean time duration for specimen obtaining process by MOOH and PHNSS and the mean time duration for specimen reading process by a cytoscreener.

Data were entered by using SPSS version 20. Clients’ perceptions of new HPV/DNA screening implementation were expressed by percentages. The overall prevalence of cervicovaginal HPV infection with 95% confidence intervals and subgroup analysis by serotypes were carried out. HPV/DNA screen positive follow-up results were expressed by percentages. HPV/DNA screening operational and technical feasibility indicators were calculated.

## Results

Respondents (91.1%, *n* = 836) were women, who attended for an HPV test, and non-respondents (8.9%, *n* = 82) were women, who had an interview but did not attend for an HPV test. A total number of 822 women were subjected to HPV/DNA specimen collection after excluding 14 women at the clinic setting (Flow diagram as an additional file 3). There was a significant difference (*p* < 0.001) between respondents and non-respondents according to nationality and religion. There was no significant difference (*p* > 0.05) between respondents and non-respondents according to the occupational status of women. The majority of women, who participated in the study were Sinhala (97.1%, *n* = 812) and Buddhist (97%, *n* = 811), while the non-participation among Muslims (11%, *n* = 9) and Islamic (11%, *n* = 9) women were high. Working women population were higher among non-respondents (30.5%, *n* = 25) than respondents (25.2%, *n* = 211) (Table 1).

**Table 1:** Distribution of non-respondents and respondents according to nationality, religion, and occupational status

Characteristics	Non-respondents		Respondents		
**1.Nationality**	**Number(n)**	**Percentage % of women**	**Number**	**Percentage%**	**Significance (*p*** **value)**
Sinhala	65	79.3	812	97.1	0.000
Tamil	08	9.7	14	1.7	
Muslim	09	11.0	10	1.2	
**2. Religion**					
Buddhism	64	78.0	811	97.0	0.000
Catholic	02	2.5	09	1.1	
Hindu	07	8.5	06	0.7	
Islam	09	11.0	10	1.2	
**Occupational status**					
Working women	25	30.5	211	25.2	0.299
Non-working women	57	69.5	625	74.8	
**Total**	**82**	**100.0**	**836**	**100.0**	

Clients’ perceptions were highly positive for the HPV/DNA screening test procedure (99.9%, *n* = 821) and 99.6% (*n* = 819) of clients were mentioned that the HPV/DNA screening test should be incorporated into the National Cervical Cancer Screening programme in Sri Lanka (Table 2). The majority of participants (99.5%, *n* = 818) were aware of screen positive follow-up of the HPV/DNA screening test and according to them the possibility to repeat a conventional pap test within 6 weeks in the case of HPV/DNA test positive result was 100% (*n* = 822) (Table 3).

**Table 2:** Distribution of participants according to perceptions related to HPV/DNA screening implementation

Perception	Number of women(n)	Percentage %
**1.Awareness about the HPV/DNA test**		
Clear	811	98.7
Not clear	11	1.3
**2. The convenience of the HPV/DNA test**		
Convenient	814	99.0
Not convenient	08	1.0
**3. Field staff performance**		
Satisfactory	821	99.9
Non-satisfactory	01	01
**4. Clinic staff performance**		
Satisfactory	819	99.6
Non-satisfactory	03	0.4
**5.An idea towards HPV/DNA test implementation**		
Positive	821	99.9
Negative	01	0.1
**6. HPV/DNA test should be incorporated into the National cervical cancer screening programme in Sri Lanka**		
Yes	819	99.6
No	03	0.4
**Total**	**822**	**100.0**

**Table 3:** Distribution of participants according to perceptions related to HPV/DNA screen positive follow-up

Perception	Number of women	Percent %
**1.Awareness about the positive follow-up**		
Yes	818	99.5
No	04	0.5
**2. Possibility to repeat a pap test within 6 weeks**		
Yes	822	100
No	0	0
**3. The convenience of the screen positive follow-up**		
Yes	804	97.8
No	18	2.2
**4. Trust regarding the confidentiality of reports**		
Yes	822	100.0
No	0	0.0
**Total**	**822**	**100.0**

Overall prevalence of the cervicovaginal high risk HPV serotype infection among 35- year age cohort ever-married women in Kalutara district was 6.2% (95%CI: 6.18–6.22%), while the prevalence of the high risk HPV serotype infection 16, 18 and 12 pooled risk (31, 33, 35, 39, 45, 51, 52, 56, 58,59, 66 and 69) were 1.7%(*n* = 14), 0.24%(*n* = 2) and 4.14%(*n* = 34) respectively (Table 4).

**Table 4:** Distribution of participants according to cervical HPV/DNA specimen result for high-risk HPV serotypes

Cervical HPV/DNA specimen results for high risk-HPV serotypes	Number of women	Percentage%	95% CI for percentages %
Negative	771	93.8	
12 pooled positive	34	4.14	4.13–4.15
16 positive	14	1.7	1.69–1.71
18 positive	2	0.24	0.23–0.25
16 & 12 pooled positive	01	0.12	0.1–0.13
**Total**	**822**	**100.0**	

Majority of women with HPV/DNA screen-positive results (6.2%, *n* = 51) were Sinhala (92.2%, *n* = 47) and Buddhist (90.2%, *n* = 46). The educational level of screen-positive women was ≤ grade 5 in 17.6% (*n* = 9), while the income was Rs ≤ 15,000 among 25.5%(*n* = 13) of screen-positive women (Table 5).

**Table 5:** Distribution of screen-positive women according to ethnicity, religion, educational level, number of pregnancy, monthly income, and occupational status

Characteristics	Number of women (n)	Percentage%
**1.Nationality**		
Sinhala	47	92.2
Tamil	3	5.9
Muslim	1	2
**2.Religion**		
Buddhism	46	90.2
Catholic	03	5.9
Hindu	01	2
Islam	01	2
**3.Educational level**		
≤ grade 5	09	17.6
> grade 5	42	82.4
**4.Number of pregnancy**		
0	00	0
1	05	9.8
2	35	68.6
3	08	15.7
4	03	5.9
5	00	0
**5.Average monthly income**		
Rs ≤ 15,000	13	25.5
Rs > 15,000	38	74.5
**6. Occupational status**		
Working women	09	17.6
On-working women	42	82.4
**Total**	**51**	**100.0**

The overall percentage of cytological abnormality ≥ ASCUS among women who were tested positive for HPV/DNA was 29.4% (*n* = 15), while the percentage of cytology ≥ASCUS among HPV/DNA screened positive for serotypes 16 and 18, only serotypes 12 pooled high risk and serotype 16 with 12 pooled high risk was 31.25% (*n* = 5), 26.5%(*n* = 9) and 100%(*n* = 1) respectively.

The biopsy tissue abnormality was found among 80% of women (*n* = 12), who were positive for HPV/DNA with cytological abnormality ≥ ASCUS. Among women with biopsy tissue abnormality 83.3% (*n* = 10) were CIN I and CIN II, while 16.7% (*n* = 2) were CIN III.

The percentage of biopsy tissue abnormality among HPV/DNA screened positive for serotypes 16 and 18 was 31.25% (*n* = 5), while the percentage of biopsy tissue abnormality among HPV/DNA screened positive for only serotypes 12 pooled high risk and serotype 16 with 12 pooled high risk positive was 17.6% (*n* = 6) and 100% (*n* = 1) respectively (Table 6).

**Table 6:** Distribution of participants according to biopsy specimen result for high-risk HPV serotypes

Cervical HPV/DNA specimen results for high-risk HPV serotypes	Number of women positive for high- risk HPV serotypes	Number of women positive for biopsy	Percentage %
12 pooled positive	34	06	17.6%
16 positive	14	05	35.71%
18 positive	2	0	0
16 & 12 pooled positive	01	01	100%
**Total**	**51**	**100.0**	

The coverage of the HPV/DNA screening test was 89.5%(*n* = 822). The percentage of invalid results reported was 0.12% (*n* = 1). The management adherence percentage of HPV/DNA positives for pap screening within 3 months of the initial screening was 100%(*n* = 51), while the percentage of women who attempted to get a colposcopy within 1 month of referral was 86.7% (*n* = 13).

The length of the clinic HPV/DNA specimen obtaining procedure per client (minutes) at a community WWC by a Medical Officer (MO) and Public Health Nursing Sister (PHNS) was 10 min and 13 min respectively, while the procedure meantime of the cytoscreener per specimen (including report writing) was 3.5 min. The mean time duration for specimen collection and transportation to the laboratory was 7 days, while the report delivery to the relevant MOH office following specimen collection was 13 days.

## Discussion

Well Woman Clinics are provided services related to prevention and early detection of some selected non-communicable diseases including cancers in women. In the beginning, cervical cancer screening through WWCs was based on only visual inspection of the cervix, and in 1998 cervical cancer screening based on pap smear was included in WWC services. One major drawback of the present program is, suboptimal sensitivity (53%) [9] of the pap smear to detect CIN II. HPV/DNA test has a high sensitivity to detect the viral load of CIN II [11]. Therefore to determine the feasibility of new HPV/DNA test implementation in the National Cervical Cancer Screening program is very important.

The clinical usefulness of HPV triage for women with ASCUS cytology even with the carefully validated test was limited by the fact on average 43.7% were high-risk HPV positive, while the prevalence of CIN II or worse was only 5.1%, in a study conducted in the United States [15]. Even if the combined testing with HPV/DNA test and pap test would reach 100% sensitivity, performing both tests doubles the number of tests and ultimately the number of colposcopy referrals would be increased, which would not be suitable for resource constraint settings. Therefore, in the present study, the feasibility of new HPV/DNA test implementation as a primary cervical cancer screening method (HPV/DNA test with cytology triage for ≥ASCUS) was assessed, since HPV testing has a higher sensitivity than pap testing.

High positive client perceptions for the HPV/DNA screening and it’s positive follow-up indicated adequate community awareness, field, and clinic staff performance in the district. The overall prevalence of high-risk cervicovaginal infection was 6.2%. When the proportion of women tested positive for high-risk HPV/DNA in a country is at least 1%, it indicates a good quality standard for the HPV/DNA screening test as a cervical cancer screening method [16].

Follow-up of women with positive HPV/DNA screening was carried out according to the algorithm of Asia Oceania guidelines for the implementation of programs for cervical cancer prevention and control [14]. The importance of follow-up of women with positive HPV/DNA screening results was explained to all recruited women at the participant recruitment stage of the study at the field level.

All HPV/DNA screened positives were referred for a pap test at a community WWC. A positive follow-up register was strictly maintained at each MOH level to minimize the loss to follow-up. All HPV/DNA screened positives with cytology results ≥ASCUS were referred to colposcopy. HPV/DNA, pap test, and colposcopy screening facility were offered to participants free of charge. Free test reports were incentives to participants. Inadequate colposcopic facilities within the district was one of the biggest challenges ever faced in the present study. Some referred clients for colposcopy had to repeatedly visit the procedure due to rush at the clinic as that was a combined clinic. Some women were reluctant to visit the Family Health Bureau, Colombo colposcopy clinic due to the traveling inconvenience.

HPV/DNA screened positive for serotypes 16 and 18 had a greater percentage of cytological abnormalities ≥ ASCUS (31.25%, *n* = 5), while the percentage of HPV/DNA screened positive for serotypes 12 pooled high risk was 26.5%(*n* = 9). Human papillomavirus serotypes 16 and 18 had a greater absolute risk of biopsy tissue abnormality (31.25%, *n* = 5) therefore the carcinogenicity of these 2 serotypes were high, while the percentage of HPV/DNA screened positive for serotypes 12 pooled high-risk serotypes was 17.6%(*n* = 6) with a low risk of carcinogenicity, which gives a similar pattern to the world [5, 15].

HPV/DNA screening as a primary cervical cancer screening will reduce the need for pap tests as only HPV/DNA positives are referred for pap cytological triage, therefore the work burden of cytoscreeners and Consultant Histopathologists would be reduced. In addition to that, it will reduce the colposcopy burden in a country by 40–50% as only HPV/DNA screened positives with cytology result ≥ ASCUS are referred for colposcopy procedure. Moreover prolonging the screening interval is recommended for HPV/DNA screening, because of its high sensitivity [14] and therefore further reduction of work burden and associated resource-saving to the country could be achieved.

Operational and technical feasibility indicators of the new HPV/DNA screening implementation were developed based on the literature review. A panel of experts reviewed and selected indicators for the study to minimize the selection bias associated with indicators. As the first author recruited subjects after carefully applying exclusion criteria for HPV/DNA screening the coverage of the HPV/DNA screening was high (89.5%, *n* = 822). The percentage of invalid results reported was 0.12% (*n* = 1), which was a good indicator for adequate staff training for the HPV/DNA screening implementation in the district. All HPV/DNA screened positive women (*n* = 51, 6.2%) who were referred had undergone a pap test within 3 months of initial screening (*n* = 51, 100%), while out of fifteen women who were referred for colposcopy 13 were attempted for a colposcopy procedure (*n* = 13, 86.7%) within 1 month of referral, which was showed good quality standards of cervical cancer screening [16]. There was no such a long lag period between specimen collection and report delivery in HPV/DNA screening like in pap screening (6–8 weeks) as the mean time duration of specimen collection and report delivery to relevant MOH offices was only 13 days, which can provide quick interventions for the detected lesions.

HPV/DNA screening was done at the laboratory of DGH Kalutara. Run size for cobas 4800 machine was 96 format from 1 to 94 specimen plus 2 controls at once, therefore during an 8-h work shift maximum of 2 runs could be achieved per day (specimens 94 × 2 = 188). If the machine runs for 28 working days per month, annually one cobas 4800 machine can screen a maximum of 63,168 specimens, which would be adequate to screen the whole Western province 35- year age cohort women population (Colombo-24,156, Gampaha-23,906, Kalutara-12,719). According to the mid-year population estimates for 2017 in Sri Lanka, the total number of 35- year age cohort women population assumed by 1% of the total population was 214,440. One procedure cost was Rs. 2881.95(USD 15.6). Therefore the country has to spend Rs. 618 million rupees (USD 3.35 million) annually to incorporate cobas 4800 HPV/DNA screening test with “thinprep cell collection media/Liquid Based Cytology (LBC)” to screen all 35-year age cohort women population in Sri Lanka.

Simple inexpensive approaches for cervical cancer screening by cobas 4800 test in low-resource settings were successfully attempted in some other countries. In a research study, each woman was subjected to HPV/DNA specimen collection with a swab placed into a sarstedt tube and a cytobrush placed into a “thinprep cell collection media” The rates of the agreement were reported very high (> 90%) for any high-risk HPV (HR-HPV) types, HR-HPV 16, HR-HPV 18 and HR-HPV 12 high pooled risk serotypes [17].

In some countries, dry transport in dry tubes was successfully attempted [18], and found that dry transport was a feasible option for transporting HPV/DNA specimens. Further to that some other countries had tried the use of glass slide as a transport medium for HPV/DNA cervical specimen and found a good agreement for HPV/DNA detection [19], while some have successfully tested an alternative media to transport and store HPV/DNA cervical specimen, i.e.. filter paper [20], pads [21] and even mouthwash solution [22].

This study was restricted to one district out of 25 districts in Sri Lanka due to logistic constraints. The population characteristics and the public health infrastructure of the district favored the generalizability of the research findings to the whole country.

When implementing a new screening test into the National programme, in a low resource setting like Sri Lanka, needs to always concentrate on the economic feasibility and cost-effectiveness. Therefore, needs to determine the most appropriate method for Sri Lanka by assessing other alternative methods of HPV/DNA testing(i.e. dry swabs, dry transport in dry tubes, alternative media to transport, etc.) by cobas 4800 screening machine.

## Conclusion

The present study has explored, that the HPV/DNA screening test is feasible to be incorporated into the National Cervical Cancer Screening programme as a primary cervical cancer screening method. It is recommended to further assess alternative methods of cobas 4800 HPV/DNA testing to determine the most appropriate method for a resource-limited setting.

## Supplementary Information

**Additional file 1 **Interviewer Administered Questionnaire**.** Basic Information & Background characteristic of the participants, Socio-demographic characteristic of participants & Participant’s perception of the new HPV/DNA screening procedure.

**Additional file 2.** Guideline for HPV/DNA cervical specimen collection for health staff.

**Additional file 3 **Number of women recruited into the study (*n* = 918).

## Data Availability

The datasets used to analyze in this study are available at the corresponding author on reasonable request.

## Abbreviations

MOH: Medical Officer of Health
PCR: Polymerase Chain Reaction
PHNSS: Public Health Nursing Sisters
PHMM: Public Health Midwives
RA: Research Assistant
WWC: Well Woman Clinic
ASCUS: Atypical Squamous Cells of Undetermined Significance
