# Supplementary material for: The feasibility of new HPV/DNA test as a primary cervical cancer screening method among 35- years- old ever-married women in Kalutara district; a cross-sectional study
Source: BMC Public Health. 2021 Jan 13;21:131. doi: 10.1186/s12889-021-10190-4 (PMC7805031; doi:10.1186/s12889-021-10190-4)
Supplement: Supplementary file 1 — Additional file 1 Interviewer Administered Questionnaire. Basic Information & Background characteristic of the participants, Socio-demographic characteristic of participants & Participant’s perception of the new HPV/DNA screening procedure. [file 12889_2021_10190_MOESM1_ESM.docx]

**Additional files**

**Additional file-1**

**Title of data-**Interviewer Administered Questionnaire

**Description of data-**Basic Information & Background characteristic of the participants, Socio-demographic characteristic of participants & Participant’s perception of the new HPV/DNA screening procedure

**Basic Information on Background characteristics of the Participant**

1).Identification number of the participant……………………………………………………

2).MOH area…………………………………………………………………………………...

3).PHM area…………………………………………………………………………………....

4).Date………………………………………………………………………………………….

**Part 1**

**Socio-demographic information of the participant**

1. What is your age at last birthday…………………………………………………………….

Date of birth

| **Date** | **Month** | **Year** |
| --- | --- | --- |
|  |  |  |

2)Marital status

i. Married…………………………………………………………………………………………

ii. Unmarried……………………………………………………………………………………..

iii. Other(specify)………………………………………………………………………………..

3).Age at marriage……………………………………………………………………………….

4).Duration of Marriage…………………………………………………………………………

5). What is your ethnicity

i. Sinhalese

ii. Tamil

iii. Moor

iv. Other (Specify)………………………………………………………………………………

6).What is your religion

i. Buddhist

ii. Catholic/Christian

iii. Hindu

iv. Islam

v. Other (specify)……………………………………………………………………………….

7). Duration of the stay at present MOH area……………………………………………………

8). Permanent residence…………………………………………………………………………..

9). District of the permanent residence…………………………………………………………..

**Part 2**

**Participant’s perception of the new HPV/DNA screening procedure**

1. Do you have a proper idea about the reason for conducting this screening procedure. Yes/No

2.Do you feel comfortable with the new screening test

Yes/No

3.If this screening test result is positive do you know you have to undergo a pap screening test.

Yes/No

4.Is it feasible for you to undergo pap smear screening within 6 weeks if the HPV/DNA screening test results positive

Yes/No

5. Do you satisfied with the privacy of the HPV/DNA procedure at the clinic setting.

Yes/No

6. Do you trust the relevance of this HPV/DNA screening test to Sri Lankan women. Yes/No

7.Do you believe this method should be incorporated in National Cervical Cancer screening

Program.

Yes/No

8.Do you have any unanswered questions about this screening test. Yes/No

9.Do you trust the confidentiality of information provided by you and screening results.

Yes/No

10. Satisfaction with the field staff performances regarding the new screening technique implementation.

Yes/No

11.Satisfaction with the clinic staff’s performances regarding the new screening technique.

Yes/No

12.Any conflict of interest regarding this new screening technique.

Yes/No
