# Supplementary material for: The feasibility of new HPV/DNA test as a primary cervical cancer screening method among 35- years- old ever-married women in Kalutara district; a cross-sectional study
Source: BMC Public Health. 2021 Jan 13;21:131. doi: 10.1186/s12889-021-10190-4 (PMC7805031; doi:10.1186/s12889-021-10190-4)
Supplement: Supplementary file 2 — Additional file 2. Guideline for HPV/DNA cervical specimen collection for health staff. [file 12889_2021_10190_MOESM2_ESM.docx]

**Additional file 2**

**Guideline for HPV/DNA cervical specimen collection for health staff**

1. The client should lay on the bed at the lithotomy position the same the waylaid on to the pap smear collection.
2. Insert the Cusco’s speculum according to the guideline by Gamage et al., 2012 [11].
3. **E**xcessive secretions are collected from the uncleaned cervix using a swab.
4. Insert central bristles of the broom-like device into the cervical canal. Allow shorter bristles to fully contact the ectocervix.
5. Gently collect cervical smear from the squamocolumnar junction of the cervix using the broom-like device, by rotating 5 times in a clockwise direction.
6. Gently remove the broom-like device and place in it the provided container with cell collection media/thinprep solution/LBC.
7. Push the broom-like device against the bottom of the vial 10 times forcing the bristles to bend apart. Swirl the broom vigorously after pushing.
8. Tighten the vial cap until the black torque line passes the black line on the vial.
9. Barcode container with accurate identification number/Well Woman Clinic register number.
10. Transport to the laboratory at room temperature as soon as possible. In a delay of transport, specimens to be stored at the bottom level of a normal refrigerator (2c˚-8c˚).
