# Supplementary material for: The feasibility of new HPV/DNA test as a primary cervical cancer screening method among 35- years- old ever-married women in Kalutara district; a cross-sectional study
Source: BMC Public Health. 2021 Jan 13;21:131. doi: 10.1186/s12889-021-10190-4 (PMC7805031; doi:10.1186/s12889-021-10190-4)
Supplement: Supplementary file 3 — Additional file 3 Number of women recruited into the study (n = 918). [file 12889_2021_10190_MOESM3_ESM.docx]

**Additional file 3**

**Number of women recruited into the study (n=918)**

Number of women who attended the clinic Number of women not attended

for HPV/DNA specimen collection **(n=836)** to the clinic for HPV/DNA specimen

collection**(n=82)**

Number of women excluded at a clinic setting

**(n=14)**

Total number of women subjected to HPV/DNA

cervical specimen collection **(n=822)**
